# Cooperation between the human T-cell leukemia virus type-1 p30(II) protein and host cellular factors during oncogenic transformation and retroviral carcinogenesis

**DOI:** 10.1186/1742-4690-11-S1-P95

**Published:** 2014-01-07

**Authors:** Megan Romeo, Braden Barnett, Janice Kim, Paige Seeker, Jeffrey He, Alexander McRae, Fahmida Musharof, Hayley Heatley, Afrida Faria, Jennifer Tran, Jordan Milam, Robert Harrod

**Affiliations:** 1 Laboratory of Molecular Virology, Department of Biological Sciences and the Dedman College Center for Drug Discovery, Design & Delivery, Southern Methodist University, Dallas, Texas, USA

## 

The human T-cell leukemia virus type-1 (HTLV-1) pX-encoded accessory factors, p30II and Hbz, suppress proviral gene expression and help to maintain latency as a prerequisite for the development of adult T-cell leukemia/lymphoma (ATLL). Our laboratory is studying how these viral proteins influence host cellular gene expression and signaling which may contribute to hematological disease progression. There are five clinically-defined stages of HTLV-1-associated disease (pre-ATLL, smoldering T-cell leukemia, chronic T-cell leukemia, acute T-cell leukemia, and non-Hodgkin’s T-cell lymphoma) and the transitional molecular events which lead to acute/lymphoma-stage disease are not well understood. We have previously shown that p30^II^ interacts with the c-MYC oncoprotein and enhances c-MYC-dependent transactivation and oncogenic potential by stabilizing recruitment of the TIP60 acetyltransferase to p30^II^/c-MYC/TIP60 transcription complexes. We now extend these findings by demonstrating that p30^II^ induces acetylation of the c-MYC protein. Acetylation-defective Lys --> Arg substitution mutants of c-MYC (R5, K323R/K417R) are impaired for oncogenic foci-formation by p30^II^/c-MYC. Acute/lymphoma-stage ATLL clinical isolates frequently exhibit overexpression of c-MYC, due to 8q24 chromosomal translocations and/or c-myc gene amplification, and the HTLV-1-transformed T-cell-lines HuT-102 and MJG11 display significant acetylation of c-MYC. The *p*53 tumor suppressor is a downstream target of c-MYC and, coincidentally, most ATLL leukemic lymphocytes contain high intracellular levels of wildtype p53. Our recent studies demonstrate that p30^II^ activates p53 and induces the expression of p53-dependent anti-apoptotic genes which could promote oncogene-activation and contribute to ATLL tumorigenesis. These findings as well as a novel role for p30^II^ in the long-term proliferation of lentiviral-p30^II^-transduced primary human T-lymphocytes will be discussed.

